# In Vitro Production of Galactooligosaccharides by a Novel β-Galactosidase of *Lactobacillus bulgaricus*

**DOI:** 10.3390/ijms232214308

**Published:** 2022-11-18

**Authors:** Alexander Arsov, Ivan Ivanov, Lidia Tsigoriyna, Kaloyan Petrov, Penka Petrova

**Affiliations:** 1 Institute of Microbiology, Bulgarian Academy of Sciences, 1113 Sofia, Bulgaria; 2 Institute of Chemical Engineering, Bulgarian Academy of Sciences, 1113 Sofia, Bulgaria

**Keywords:** β-galactosidase, *Lactobacillus bulgaricus*, prebiotic

## Abstract

β-galactosidase is an enzyme with dual activity and important industrial application. As a hydrolase, the enzyme eliminates lactose in milk, while as a trans-galactosidase it produces prebiotic galactooligosaccharides (GOS) with various degrees of polymerization (DP). The aim of the present study is the molecular characterization of β-galactosidase from a Bulgarian isolate, *Lactobacillus delbrueckii* subsp. *bulgaricus* 43. The sequencing of the *β-gal* gene showed that it encodes a new enzyme with 21 amino acid replacements compared to all other β-galactosidases of this species. The molecular model revealed that the new β-galactosidase acts as a tetramer. The amino acids D207, H386, N464, E465, Y510, E532, H535, W562, N593, and W980 form the catalytic center and interact with Mg^2+^ ions and substrate. The *β-gal* gene was cloned into a vector allowing heterologous expression of *E. coli* BL21(DE3) with high efficiency, as the crude enzyme reached 3015 U/mL of the culture or 2011 U/mg of protein. The enzyme’s temperature optimum at 55 °C, a pH optimum of 6.5, and a positive influence of Mg^2+^, Mn^2+^, and Ca^2+^ on its activity were observed. From lactose, β-Gal produced a large amount of GOS with DP3 containing β-(1→3) and β-(1→4) linkages, as the latter bond is particularly atypical for the *L. bulgaricus* enzymes. DP3-GOS formation was positively affected by high lactose concentrations. The process of lactose conversion was rapid, with a 34% yield of DP3-GOS in 6 h, and complete degradation of 200 g/L of lactose for 12 h. On the other hand, the enzyme was quite stable at 55 °C and retained about 20% of its activity after 24 h of incubation at this temperature. These properties expand our horizons as regards the use of β-galactosidases in industrial processes for the production of lactose-free milk and GOS-enriched foods.

## 1. Introduction

Galactooligosaccharides (GOS) are among the rare carbohydrates that fully satisfy the requirements to be prebiotic [1]. They cannot be hydrolyzed in the human upper gastrointestinal tract [2], cause a significant reduction in the number of harmful bacteria, enhance bifidobacterial growth in the colon [3,4], and induce a pronounced beneficial effect on the health of the consumer [5,6]. Besides gut status improvement [7], GOS consumption benefits calcium absorption and bone mineralization [8], relieves lactose intolerance and prevents constipation [9], alleviates atopic dermatitis [10], regulates lipid metabolism, prevents obesity [11,12], and diminishes the risk of colorectal cancer [13].

Prebiotic GOS include the non-digestible oligomers containing β-linked galactose residues, excluding the disaccharides lactose and melibiose [14,15]. They vary in both chain length and the way the monomer units are linked, typically containing terminal glucose and 2 to 8 galactose residues. In nature, GOS are present in small amounts in cow, camel, and human milk [16,17,18]; and in high amounts in the milk of kangaroo [19].

Due to the high demand for infant milk and prebiotics formulae preparations, the commercial production of GOS increases by 6% annually [2], and the global market profit is expected to reach USD 10.55Bby 2025 [20]. GOS are commercially obtained by chemical methods, which are not preferable because they generate unwanted additional compounds [21], or via enzymatic synthesis from lactose by β-galactosidases of fungal or bacterial origin. The most frequently used enzymes are those of *Kluyveromyces lactis* and *Aspergillus oryzae*, producing mainly β-(1→6)-linked GOS, and that of *Bacillus circulans* for β-(1→4)-linked GOS. However, it was observed that β-galactosidases from several probiotic species form distinct GOS structures and grow more efficiently on the oligosaccharides that are produced by their own β-galactosidases, compared to commercial GOS [22]. In addition, the enzymes of lactobacilli and bifidobacteria have recently received special attention in terms of transgalactosylation activity, since they have the propensity to catalyze this reaction [23].

According to the CAZy database [24], β-galactosidases (EC 3.2.1.23) are structurally diverse enzymes. Based on their amino acid sequences, hydrophobic clusters, reaction mechanism, and the conservation of catalytic residues, they are classified into GH1, GH2, GH35, and GH42 families. All *Lactobacillus* species contain β-galactosidases of the GH2 family, described in *L. acidophilus*, *L. coryniformis*, *L. johnsonii*, *Lactiplantibacillus plantarum*, *Limosilactobacillus reuteri*, and *Latilactobacillus sakei*. They are encoded by the genes *lacL* and *lacM* and form heterodimers of the LacLM type [25,26]. In contrast, the β-galactosidases of *L. helveticus* and *L. delbrueckii* subsp. *bulgaricus* (*L. bulgaricus*) are homomeric, of LacZ type, and far less investigated [15,27].

Our recent work showed that a newly isolated indigenous *L. bulgaricus* strain 43 can spontaneously form high amounts of GOS with DP3 and DP4 in yogurt [28]. In addition, GOS structures contained β-(1→4) bonds, which is quite unusual for *L. bulgaricus*. Therefore, the present work aimed to investigate the responsible enzyme by its gene sequencing, heterologous expression, and biochemical characterization, and to reveal its potential to produce GOS.

## 2. Results

### 2.1. Gene Sequencing and Molecular Structure of the New β-Galactosidase of L. bulgaricus 43

**Figure 1:**
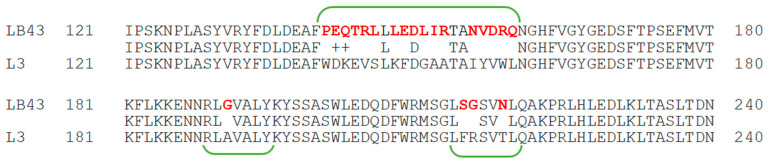
Amino acid sequence comparison of the region possessing the lowest homology with other β-galactosidases of *L. bulgaricus*. The presented comparison is with the sequence of a well-studied β-galactosidase of *L. bulgaricus* strain L3 [28], NCBI GenBank number ACE06986.

Based on the *lacZ* gene sequence of the referent strains *L. delbrueckii* subsp. *bulgaricus* DSM 20080 and ATCC 11842, a primer pair targeting the gene in *L. bulgaricus* 43 was designed. Thus, a DNA fragment (3027 bp) was PCR-amplified, sequenced, and deposited in NCBI GenBank with accession number OP617280. The nucleotide sequence comparison showed 98.32% homology with *lacZ* of the referent *L. delbrueckii* ssp. *bulgaricus* ATCC 11842, and the deduced protein sequence contained 97.52% identical amino acids with those of the β-galactosidases of the species. The lowest identity possessed the sequence regions 140–160 and 214–220, as 21 amino acid substitutions were observed (shown in red in Figure 1).

**Figure 2:**
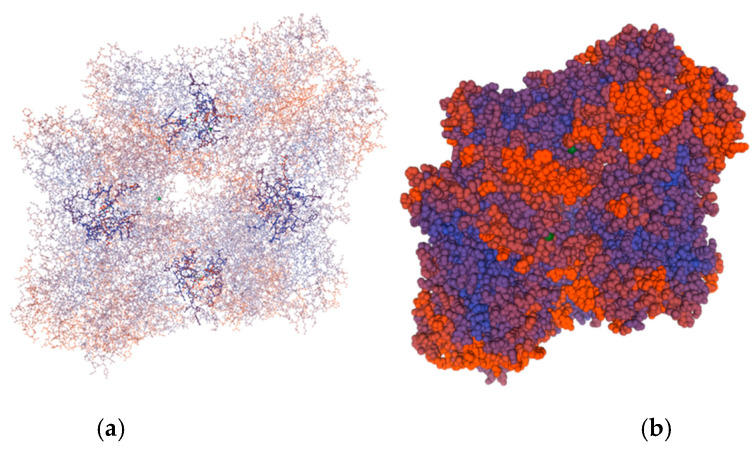
Three-dimensional model of β-galactosidase of *L. bulgaricus* strain 43 made by SWISS-MODEL Workspace [29]. (**a**) “Ball and stick” presentation of the chains revealing the formation of four active centers around the substrate; (**b**) surface model of the tetramer.

The analysis of the protein sequence of the β-galactosidase molecule by SWISS-MODEL [29] revealed that the enzyme is a homo-tetramer (Figure 2). Each of the four chains contains 1008 amino acids and has a calculated molecular weight of 114.17 kDa.

**Figure 3:**
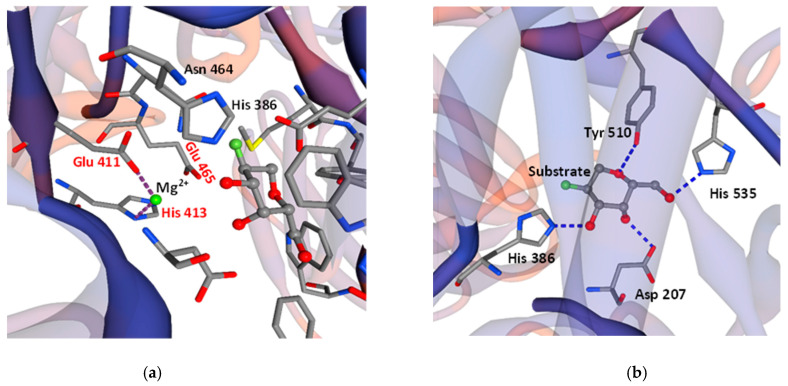
SWISS-MODEL Workspace/GMQE prediction of interactions between amino acids in the active center of β-galactosidase of *L. bulgaricus* strain 43. (**a**) According to the model, glutamates (Glu 411, Glu 465) and His 413 contact metal cations; (**b**) two histidine residues (His 386, His 535), Asp 207, and Tyr 510 form hydrogen bonds with the substrate as a part of the active center.

Falling within 4Å, 12 residues are essential for the active center formation and substrate binding: Asp207, His386, Asn464, Glu465, Met509, Tyr510, Glu532, His535, Trp562, Phe590, Asn593, and Trp980. The catalytic center is formed by Glu465 and Glu532 (which act as nucleophile and acid/base catalysts), and Tyr510, which donates a proton to Glu532 to attack the substrate. Residues Trp562, Phe590, and Trp980 most probably act as analogs of Trp570, Phe616, and Trp593 of the β-galactosidase of *Bacillus circulans* ATCC 31382, i.e., they form the aromatic pocket, which determines the linkage preference and product size. Two histidine residues (His386, His535), Asp 207, and Tyr510 form hydrogen bonds with the substrate as a part of the active center (Figure 3).

### 2.2. Cloning, Heterologous Expression in E. coli Strain BL21(DE3), and Purification of β-Galactosidase of L. bulgaricus 43

**Figure 4:**
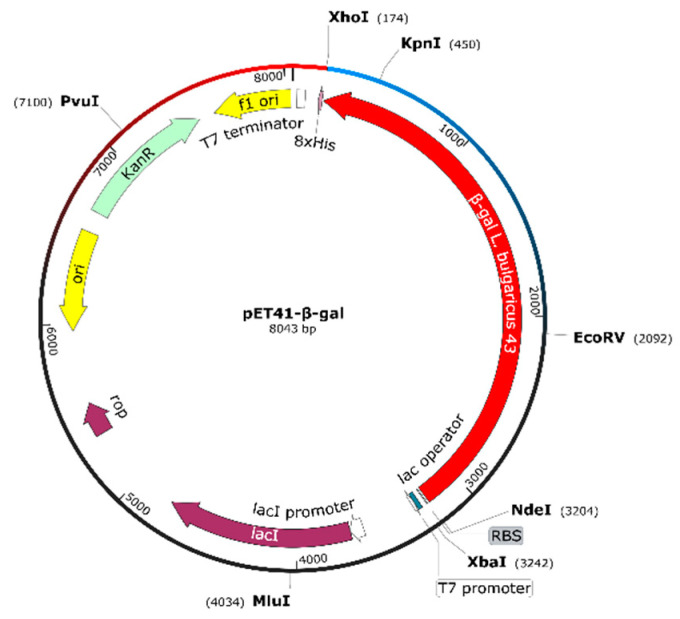
Physical map of the construct pET41-*β-gal* based on vector pET41b(+) and PCR-amplified fragment containing the β-galactosidase gene of *L. bulgaricus* strain 43.

The β-gal gene from *L. bulgaricus* 43 (3024 bp) was amplified by a PCR with appropriate primers. The obtained fragment was cloned into pET-41b(+) in *Xho*I and *Nde*I sites (replacing the gst gene of the vector), under T7 inducible promoter control, and fused to a His-tag sequence. The proper clone selection was performed using Escherichia coli HST08 strain (STELLAR^TM^ competent cells), and the recombinant construct pET41-β-gal (8043 bp, Figure 4) with the confirmed sequence was introduced into *E. coli* BL21(DE3) cells via electroporation.

The overexpression of the recombinant protein in *E. coli* was achieved by T7 promoter induction with 1 mM of isopropyl β-d-1-thiogalactopyranoside (IPTG) after the culture reached OD_600_ 0.8 at 37 °C. About 24 h after the induction, the cells reached the highest β-galactosidase activity of 3015 ± 28 U/mL of the bacterial culture (2011 ± 16 U/mg of protein of the crude enzyme), by the standard assay with substrate *o*-nitrophenol-β-D-galactopyranoside (ONPG), at pH 7.0 and 37 °C.

**Figure 5:**
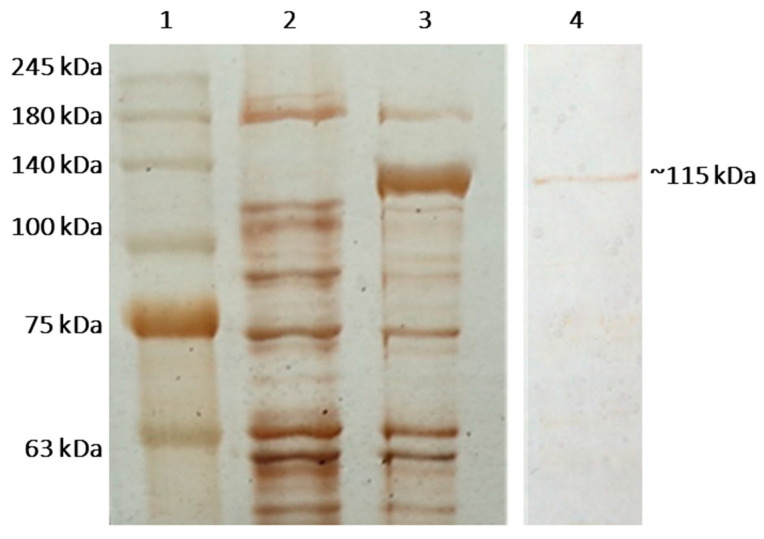
Overexpression and purification of recombinant β-galactosidase of *L. bulgaricus* 43 in *E. coli* BL21(DE3), demonstrated by SDS-PAGE in 10% separating gel after silver staining. Legend: (1) Perfect^TM^ Tricolor Protein Ladder; (2) crude extract of the cells of untransformed *E. coli* BL21(DE3) as control; (3) crude extract from *E. coli* BL21(DE3) cells, bearing pET-41-β-gal and induced with 1 mM IPTG for 24 h; (4) the purified enzyme β-galactosidase.

The SDS-PAGE analysis of the lysate showed the presence of a band of approximately 115 kDa (Figure 5), which was missing in the control *E. coli* BL21(DE3).

Further purification of the enzyme was performed based on its 8x histidine tag by affinity chromatography, using gravitation columns containing Ni-sepharose (His Gravi Trap^TM^). The optimization of the elution with various imidazole concentrations from 100 to 1000 mM showed that the most efficient is 150 mM of imidazole (Figure 5). However, the purification of the target protein was accompanied by great losses during the elution of unwanted proteins, which is why further experiments were conducted with the crude enzyme.

### 2.3. Optimal Activity of the Recombinant β-Galactosidase

**Figure 6:**
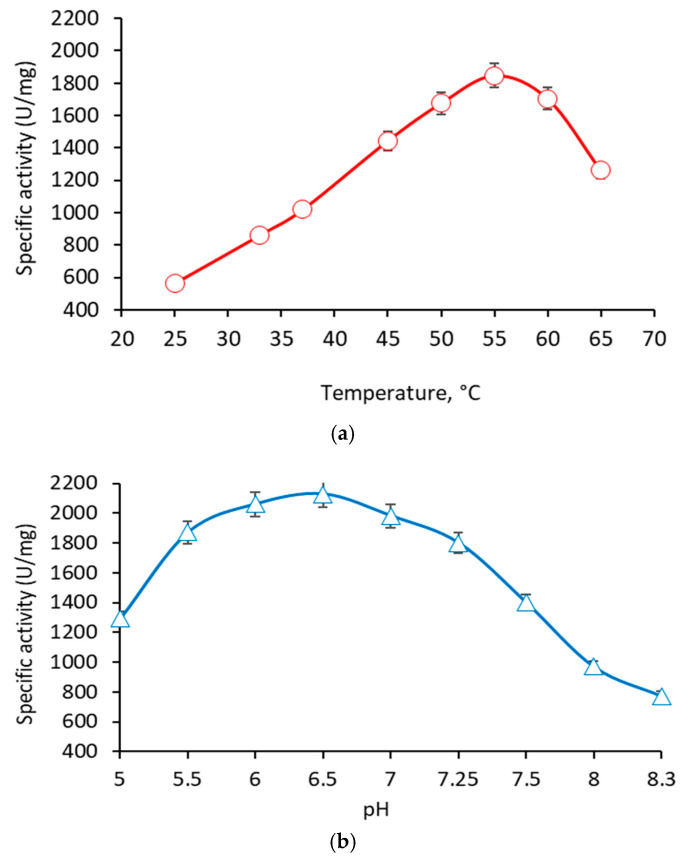
Influence of temperature (**a**) and pH (**b**) on the enzyme activity of the recombinant β-galactosidase from *L. bulgaricus* 43.

The recombinant β-galactosidase showed optimal hydrolytic activity at 55 °C (Figure 6a). Less than 25% loss of activity was observed in the range from 45 to 65 °C. At 37 °C, the optimal activity was almost halved (51.7%). The enzyme possessed a relative thermostability, since about 20% of its activity was retained after 24 h at 55 °C, and about 15% after 60 min at 60 °C. Regarding pH, the recombinant enzyme showed optimal activity at pH 6.5 (Figure 6b). Less than 25% loss of activity was observed in the relatively wide range from pH 5.5 to 7.5. The activity dropped sharply at pH 5 and pH 8, reaching only 61 and 46%, respectively, of the optimal values.

### 2.4. Influence of the Cations on the Enzyme Activity of the Recombinant β-Galactosidase

**Figure 7:**
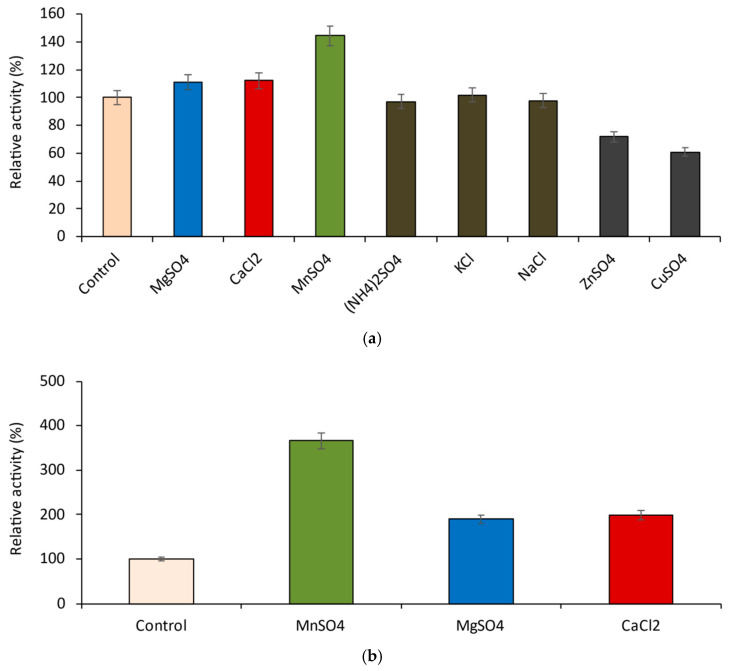
Influence of various cations on the activity of the recombinant β-galactosidase at 10 mM of salts; (**a**) assay conditions: 37 °C and pH 7.0; (**b**) assay conditions: 55 °C and pH 6.5.

**Figure 8:**
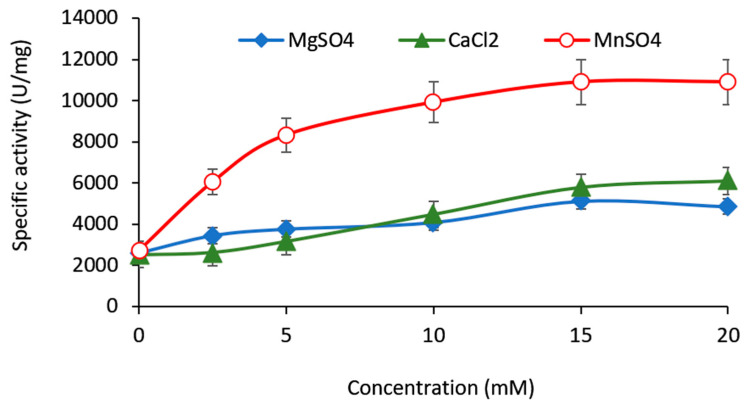
Dose-dependent influence of Mg^2+^, Ca^2+^, and Mn^2+^ on the activity of the recombinant β-galactosidase.

Eight different cations, ammonium, mono- and bivalent metal ions, were investigated for their possible influence on the hydrolytic activity of the recombinant β-galactosidase with ONPG as substrate (Figure 7 and Figure 8).

Ammonium ion (NH_4_)^+^, K^+^ and Na^+^ (as 10 mM salts) did not affect the enzyme, while the same concentrations of Cu^2+^ and Zn^2+^ slightly inhibited it (Figure 7a). Mn^2+^, Mg^2+^, and Ca^2+^ were selected for further study, as they act as enzyme enhancers. Under optimal conditions, manganese caused the most potent increase in the enzyme activity (more than 3.5 times, compared to the control), while the samples with magnesium and calcium showed almost twice (90 and 98%, respectively) higher activity (Figure 7b). Dose-dependent studies with all three salts revealed that they reached similar plateaus at 15 mM. However, this concentration proved unproductive for GOS synthesis in vitro (data not shown); hence, 10 mM was chosen for further experiments (Figure 8).

### 2.5. In Vitro GOS Production by the Recombinant β-Galactosidase

**Figure 9:**
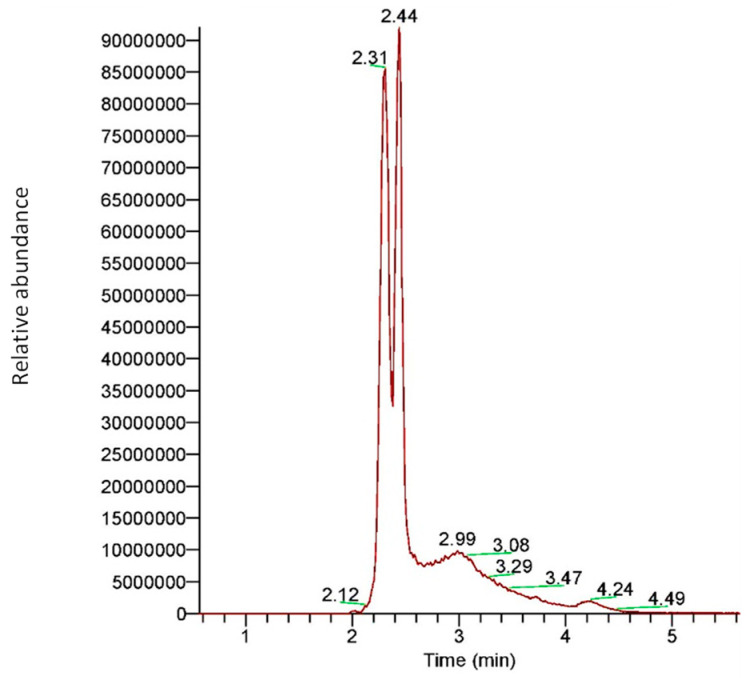
Extracted ion chromatogram for galactooligosaccharides with DP3 (trisaccharides) obtained by β-galactosidase of *L. bulgaricus* 43. The peak at 2.31 min corresponds to GOS with β-(1→3) bond, while the peak at 2.44 min corresponds to GOS with β-(1→4) linkage.

A mass-spectral qualitative study of the GOS produced by the β-galactosidase of *L. bulgaricus* 43 from 40 g/L of lactose revealed that the enzyme predominantly produces GOS with DP3. Two types of DP3 molecules were detected in approximately equal amounts. As presented in Figure 9, these structural isomers contain galactose residue connected with lactose by either β-(1→3) or β-(1→4) linkages. The HRAMS analysis (as well as HPLC results for the heterologously expressed enzyme) showed that GOS with DP4 were produced in negligible amounts.

**Figure 10:**
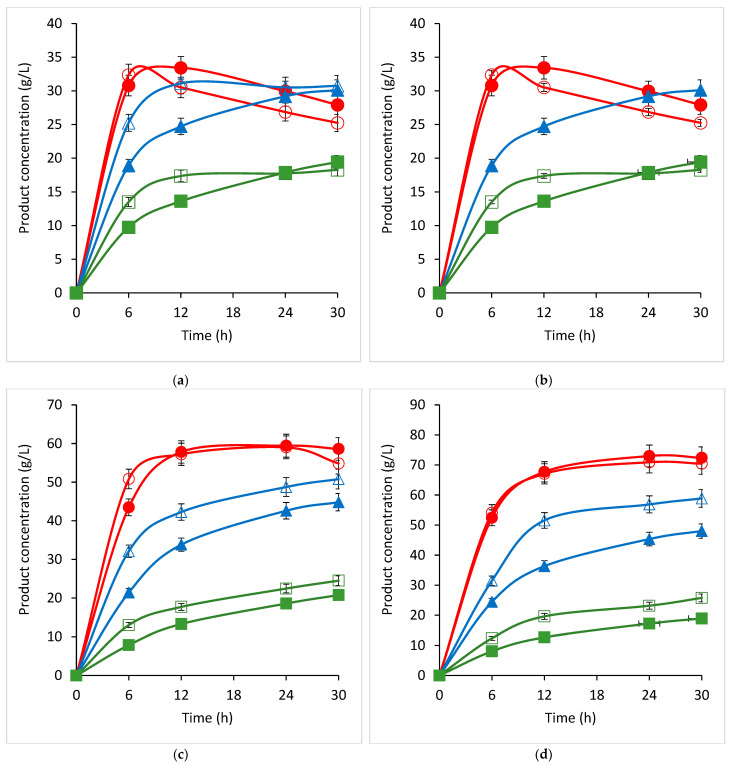
Time profiles of products formation by the action of the recombinant β-galactosidase with different concentrations of lactose: (**a**) 80 g/L; (**b**) 120 g/L; (**c**) 160 g/L; (**d**) 200 g/L of lactose. Legend: red, DP3; blue, glucose; green, galactose. Opened symbols, lactose + 1 mM of MgSO_4_; closed symbols, lactose + 10 mM of MgSO_4_, 10 mM of MnSO_4_, and 10 mM of CaCl_2_. Three independent trials were performed.

To establish the potential of the recombinant β-galactosidase to produce GOS, different concentrations of lactose were incubated with 40 U/mL of crude recombinant enzyme, and the enzymatic conversion products were subjected to an HPLC analysis. The ability of the recombinant β-galactosidase for GOS production in vitro is considerable and dose-dependent concerning the substrate (Figure 10). It reached peak values of 70.91 g/L of GOS with DP3 after 12–24 h of incubation with a 200 g/L initial lactose concentration (Figure 10d). This value was not only considerably higher than the values at the same time point for 160 and 120 g/L of lactose (20 and 67%, respectively), but it also remained more stable for the next six hours. Lower concentrations of the substrate increased the speed of the enzyme reaction; at 120 and 80 g/L lactose, the DP3 GOS peak was reached after 12 or even only 6 h, respectively.

The effects of metal cations on the transgalactosylation activity of the recombinant β-galactose certainly differed from those observed on their hydrolytic activity assayed with ONPG. The positive combined effect of Mn^2+^, Mg^2+^, and Ca^2+^ for GOS production was pronounced at 80 g/L of lactose, where a consistent increase in the DP3 GOS production was observed (about 10%). However, at higher lactose concentrations (120–200 g/L), this effect disappeared. Higher concentrations of salts of Mn^2+^, Mg^2+^, and Ca^2+^ (15 mM) proved to have an inhibitory effect on the DP3 GOS production, both separately and when combined (data not shown).

## 3. Discussion

The present article is devoted to the sequencing of the gene encoding a novel β-galactosidase in *L. bulgaricus* strain 43, the modeling of a three-dimensional structure of the enzyme, and its biochemical characterization. Current data on β-galactosidases of *L. bulgaricus* are rather scarce, as until ten years ago some strains were thought to synthesize a truncated enzyme [30], and only with the accumulation of data of whole genome sequencing did the sequence of the responsible genes become clear. The *β-gal* gene of *L. bulgaricus* 43 encodes a new enzyme with 21 amino acid substitutions compared to the previously known ones. These substitutions are essential for the properties of the enzyme, as they are located in close proximity to the active site at the N-terminus. The 3D model of the molecule revealed that the enzyme of strain 43 acts as a tetramer, which is reported for the first time for the species *L. bulgaricus* and is most likely due to the large differences in the amino acids in region aa 140—aa 220. According to Weber and Schneider [31], protein dimers are stabilized by amino acids rich in amino acids with small side chains, such as Gly, Ala, or Ser, and many of them contain Gx-like motifs. In the sequence of the β-Gal of *L. bulgaricus* 43, three new such aa residues appear—two glycines at positions 191 and 215 and one serine at position 214. Considering that in *E. coli* the active centers of the enzyme are formed by the chains’ interactions [32], and that “SV”, “GV”, and “SG” motifs stabilize the dimeric structures [31], these substitutions are most likely the reason for the tetrameric structure of the enzyme in strain *L. bulgaricus* 43 and its high activity. The structure of the catalytic site of β-Gal is in agreement with the bioinformatic analysis of Bultema et al. [33], who studied the structure of *B. circulans* BgaD, a similar retaining-type glycosidase of glycoside hydrolase family 2 (GH2). Compared to BgaD, the enzyme of *L. bulgaricus* 43 contains conserved residue Glu 532, which is the nucleophile in the enzyme reaction; the role of acid/base catalyst in β-Gal is played by Glu 465 (Glu 447 in *B. circulans*). Similarly, two histidine residues, His386 and His535, are present in the active site. Notably, like β-Gal, BgaD of *B. circulans* forms trisaccharides with β-(1→4)-linkages as a major transgalactosylation product [34]. One difference, however, is that BgaD of *B. circulans* is Mg^2+^-independent, while β-Gal of *L. bulgaricus* 43 needs Mg^2+^ (at least 0.5 mM) for its activity, because this bivalent metal cation acts as a cofactor in the catalytic site. The magnesium ion could be frequently substituted by Mn^2+^, which our results confirmed by the 366% increased enzyme activity of β-Gal in the presence of 10–15 mM of MnSO_4_. This strangely differs from other *L. bulgaricus* β-galactosidases. For example, Nguyen et al. [27] cloned and overexpressed the *lacZ* gene from *L. bulgaricus* DSM 80021 in *L. plantarum*, but the resulting enzyme was activated by K^+^ and Na^+^ (more than 5 and 10 times, respectively, at 10 mM) and inhibited by Ca^2+^ and Mg^2+^ (more than 60% at 10 mM), a stark contrast with the β-Gal of strain 43. Similar sensitivity to metals was shown by β-galactosidases from other species, for instance, one from *L. leichmannii* 313, which is also activated by Na^+^ (five times at 10 mM) but inhibited by Ca^2+^ (almost 50%) and, interestingly, Mn^2+^ (nearly 70%, both at 10 mM), yet remains unaffected by K^+^. Considering the other parameters, the pH optimum of the last enzyme is 5.5, but with very narrow margins: more than 50% loss of activity within half a pH unit in either direction [35]. Again, this is significantly different from our enzyme, which loses the same activity over a thrice wider range of pH values. A temperature optimum of 55 °C allows GOS production at elevated temperatures, which increases lactose solubility and process productivity.

Since the enzymatic activity of β-galactosidase in *L. bulgaricus* is generally low [30], heterologous expression of the responsible gene in different microbial hosts is a preferred method for studying the enzyme and for its application in the synthesis of prebiotic GOS [27]. However, the highest recombinants’ activities reported so far are slightly above 300 U/mg (Table 1), more than six times weaker than the β-galactosidase of *L. bulgaricus* 43. Recombinant enzymes derived from other *Lactobacillus* spp., such as *L. helveticus* and *Limosilactobacillus. fermentum*, have raised the bar to almost 500 U/mg, but no more than that. Many of these studies have reported significant substrate-related differences, the activity with ONPG being from 3 to 40 times higher than that obtained with lactose. This discrepancy should be taken into account when downstream applications of the enzyme are considered. The same may be said about reports that recombinant enzymes with His-tag habitually show 20-30% lower activity [27]. As far as GOS yields are concerned, *L. bulgaricus* strains have the edge over other lactobacilli, though even with them, half of the total sugar content appears to be the limit. This status quo may change, in time, when various enzyme enhancers are studied in more detail than they hitherto have been. The present study is a case in point. The unique sensitivity of our β-galactosidase to metal cations opens opportunities for optimization, which should be explored in the future.

## 4. Materials and Methods

### 4.1. Bacterial Strains and Maintenance

*L. delbrueckii* subsp. *bulgaricus* strain 43 was from cow yogurt produced in the town of Smilyan in the Rhodope Mountains, Smolyan Municipality, Bulgaria. It was identified by 16S rRNA gene sequencing (NCBI GenBank accession no MG437371).

The pure bacterial culture was maintained in MRS medium at 42 °C and stored frozen at −80 °C, supplemented with 15% glycerol. For DNA isolation, the strain was grown in 50 mL of MRS broth in laboratory bottles (Isolab Laborgeräte GmbH, Eschau, Germany), at anaerobic conditions, using Anaerocult**^®^** A mini (Merck KGaA, Darmstadt, Germany).

*E. coli* HST08 strain (STELLAR^TM^ competent cells, genotype F-, *endA1*, *supE44*, *thi-1*, *recA1*, *relA1*, *gyrA96*, *phoA*, *F80d lacZD M15*, *D(lacZYA-argF) U169*, *D(mrrhsdRMS-mcrBC*), *DmcrA*, λ-) was purchased from Clontech Laboratories, Inc., A Takara Bio Company (Mountain View, CA, USA). *E. coli* BL21(DE3) strain was purchased from New England Biolabs (Ipswich, MA, USA).

Both *E. coli* strains were cultivated in Luria-Bertani (LB) medium, at 37 °C, solidified with 15% agar (HiMedia, Mumbai, India) when needed. For the transformants’ selection, kanamycin with a concentration of 50 μg/mL was used.

### 4.2. Isolation of DNA, PCR, and Sequence Analysis of β-Galactosidase Gene of L. bulgaricus 43

Total genomic DNA from *L. bulgaricus* strain 43 was extracted with the GeneMATRIX Bacteria & Yeast Genomic DNA Purification Kit (EURx**^®^**, Gdansk, Poland).

PCR amplification of the fragment (containing the *β-gal* gene) was performed in QB-96 Satellite Gradient Thermal Cycler (LKB Vertriebs GmbH, Vienna, Austria).

PCR reactions consisted of a 150 ng DNA template, 0.4 µM of primers, 12.5 μL of Premix Ex Taq Hot Start Version 2.0 (Clontech Laboratories, Inc., A Takara Bio Company (Mountain View, CA, USA), and nuclease-free water (EURx**^®^**, Gdansk, Poland) to a 25 µL final volume. The next temperature profile was as follows: initial denaturation, 3 min at 95 °C; 35 cycles: 10 s denaturation at 98 °C, 30 s annealing at 60 °C, and 1 min elongation at 72 °C; final elongation—5 min at 72 °C. A gradient of annealing temperatures from 54.2 °C to 62.7 °C showed no loss of quality in the PCR product. Two pairs of primers, listed in Table 2, were used for sequencing the complete PCR fragment (Macrogen Inc., Amsterdam, The Netherlands).

DNA fragments were visualized using gel electrophoresis on agarose (AlfaAesar, Kandel, Germany), in TAE buffer (40 mM of Tris-base, 20 mM of acetic acid, 1 mM of EDTA), and stained with SimplySafe^TM^ (EURx, Gdansk, Poland).

### 4.3. Bioinformatics Analysis

The obtained nucleotide sequences were processed by ChromasPro 2.1.10 (https://technelysium.com.au/wp/, accessed on 10 August 2022) and assembled with CAP3. The deduced amino acid sequence was received by Expasy Translate Tool (Swiss Institute of Bioinformatics). The comparison with the NCBI GenBank database was made by BLASTN, BLASTP (NCBI), and alignment by ClustalW programs (https://www.genome.jp/tools-bin/clustalw, accessed on 14 September 2022). A molecular map of the construct pET-41-β-gal was drawn with the program SnapGene**^®^** (GSL Biotech LLC).

Molecular modeling of the β-galactosidase gene of *L. bulgaricus* 43 was performed in SWISS-MODEL Workspace [29].

### 4.4. Molecular Cloning of the β-Galactosidase Gene of L. bulgaricus 43

For cloning of the *β-gal* fragment, PCR amplification was performed with a primer pair containing introduced restriction sites (Table 2). Thus, obtained in sufficient amounts, the fragment was digested with *Xho*I (Thermo Fisher Scientific, Waltham, MA, USA) and *Nde*I (New England Biolabs, Ipswich, MA, USA), and cloned into a pET-41b(+) vector (Novagen, Merck KGaA, Darmstadt, Germany) designed for high expression of recombinant proteins with a tag of eight histidine residues (8xHis).

The recombinant construct pET-41-β-gal was used for the transformation of *E. coli* HST08 Stellar^TM^ competent cells, according to Protocol PT5055-2 of the manufacturer. Successful clones were confirmed by a restriction analysis and sequencing. The correct construct was used for the transformation of *E. coli* BL21(DE3) via electroporation of competent cells, which were obtained after repeated washing with ice-cold 10% glycerol. BioRad MicroPulser (BioRad Laboratories, Hercules, CA, USA) and a pulse of 1.8 kV for 5.7 ms, using Gene Pulser**^®^** Cuvettes with a 0.1 cm electrode gap, were used for *E. coli* transformation. SOC medium (2% tryptone, 0.5% yeast extract, 10 mM of NaCl, 2.5 mM of KCl, 10 mM of MgSO_4_) with freshly added glucose (20 mM) and MgCl_2_ (10 mM) was used as a recovery medium.

### 4.5. Preparation of the Crude β-Galactosidase

A frozen stock of 100 μL of *E. coli* BL21 (DE3) cells harboring the construct pET41-*β-gal* was inoculated in 50 mL of LB medium, containing 100 μg/mL of kanamycin (AppliChem, GmbH, Darmstadt, Germany) and cultivated overnight at 37 °C. With this culture (5%, *v*/*v*) were inoculated 500 mL Erlenmeyer flasks containing 60 mL of the same medium, and the cultures were cultivated at 37 °C on a rotary shaker (130 rpm) until OD_600_ reached 0.8. Then, IPTG (isopropyl β-d-1-thiogalactopyranoside (AppliChem, GmbH, Darmstadt, Germany) with variable concentrations (0.5–1.5 mM) was added to the cultures to induce the expression of the target gene. After 24 h of cultivation, the cells were harvested and resuspended in potassium-phosphate buffer (50 mM of K_2_HPO_4_, 50 mM of KH_2_PO_4_, pH 7), then disrupted by sonication using an ultrasonic homogenizer—Bandelin Sonoplus 2070 (BANDELIN electronic GmbH & Co., KG, Berlin, Germany)—set at 20 kHz, 5 sec pulses, for 10 min in an ice bath. After centrifugation for 15 min on 12,500× *g* and 4 °C, the supernatant was decanted and stored frozen at −20 °C.

### 4.6. Purification and Visualization of β-Galactosidase of L. bulgaricus 43

The crude enzyme was purified with Ni Sepharose columns His GraviTrap^TM^ and His Buffer Kit (GE Healthcare, Uppsala, Sweden). The columns were equilibrated and washed, and the crude lysate mixed, with 20 mM of imidazole in phosphate buffer with a pH 7.4, containing 20 mM of Na_3_PO_4_ and 500 mM of NaCl. The most successful purification was achieved with 150 mM of imidazole in the elution buffer.

The purified β-galactosidase and crude lysates were subjected to SDS-PAGE (5% stacking and 10% separating gels, 150 V for 90 min). The proteins were visualized by silver staining. Perfect^TM^ Tricolor Protein Ladder (EURx, Gdansk, Poland) was used as a molecular weight marker.

### 4.7. Enzyme Activity Assay

The hydrolytic activity of the β-galactosidase was measured by the ability of the crude enzyme to hydrolyze the chromogenic substrate *o*-nitrophenol-β-D-galactopyranoside (ONPG; Sigma-Aldrich, St. Louis, MO, USA). The crude enzyme was diluted in sodium-potassium buffer (100 mM of Na_2_HPO_4_, 10 mM of KCl, 1 mM of MgSO_4_) and incubated under optimal conditions (pH 6.5, 55 °C) for 5 min, together with 15 mM of ONPG. The reaction was stopped with 1 M of Na_2_CO_3_, and the OD at 420 nm was measured versus blank with buffer instead of the crude enzyme using a Helios Omega UV-VIS spectrophotometer (Thermo Scientific Inc., Waltham, MA, USA). The amount of ONP was estimated with a standard curve of concentrations from 50 to 1000 μM. The specific enzyme activity is expressed in units per mg of total protein (U/mg). One unit (1U) is defined as the amount of enzyme that catalyzes the hydrolysis of 1 μmole of ONPG for 1 min. The total protein content of the crude lysate was estimated by the Bradford method.

### 4.8. Influence of Temperature, pH, and Cations on the Activity of β-Galactosidase

The hydrolytic activity of the β-galactosidase was measured in the range from 25 to 65 °C, and in different buffers with the pH adjusted from 5 to 8.3. The effect of eight different metal ions (Mn^2+^, Mg^2+^, Ca^2+^, Na^+^, K^+^, Zn^2+^, Cu^2+^, [NH_4_]^+^) was studied after 30 min incubation of the enzyme at 4 °C with 10 mM of MnSO_4_, MgSO_4_, CaCl_2_, NaCl, KCl, ZnSO_4_, CuSO_4_, and (NH_4_)_2_SO_4_. All other conditions of the enzyme activity assay remained unchanged.

### 4.9. In Vitro GOS Production

In vitro GOS synthesis was performed in laboratory bottles of 250 mL on a GFL 1092 rotary shaker (GFL Gesellschaft für Labortechnik GmbH, Burwedel, Germany). The process was carried out for 30 h under optimal conditions (pH 6.5, 55 °C). Mixtures of 25 mL of lactose solution (with variable concentration, in sodium-potassium buffer), crude enzyme, and 10 mM of various salts were incubated at 100 rpm in a water bath. Samples were taken at 0, 6, 12, 24, and 30 h, boiled at 95 °C for 5 min to terminate the enzyme reaction, and subjected to an HPLC analysis.

### 4.10. Analytical Techniques

GOS fractions were analyzed by liquid chromatography/mass spectrometry (LC/MS). TurboFlow**^®^** LC system and IonMax II**^®^** electro spray ionization module (Thermo Scientific Inc., Waltham, MA, USA); Atlantis T3, 3.5 μm (100 × 2.1 mm) column (Waters Co., USA) were used. The mobile phase contained A—20 mmol/L of ammonium acetate in water; B—buffer A/acetonitrile (1/9 *v*/*v*) at a flow rate of 300 μL/min and gradient: 0% B for 180 s; 0–60% B for 150 s; 60% B for 30 s; 60–0% B for 60 s; and 0% B for 5 min. A mass spectrometric analysis was carried out using a Q Exactive Hybrid Quadrupole-Orbitrap Mass Spectrometer (Thermo Scientific Inc., Waltham, MA, USA), which was equipped with a heated electrospray ionization module IonMax**^®^** (Thermo Scientific Co., Waltham, MA, USA). Full-scan spectrum over the *m*/*z* range of 100–2000 was acquired in negative ion mode at resolution settings of 140,000. The Q Exactive parameters were a spray voltage of 4.0 kV, sheath gas flow rate of 32, auxiliary gas flow rate of 10 L/min, spare gas flow rate of 3 L/min, capillary temperature of 280 °C, sample heater temperature of 300 °C, and S-lens RF level 50. Data acquisition and processing were carried out with the Xcalibur 2.4**^®^** software package (Thermo Scientific Inc., Waltham, MA, USA).

Quantitative estimations of GOS with DP3, glucose, and galactose were made using YL Instrument 9300 HPLC System (YL Instrument Co., Ltd., Anyang, South Korea), RI detector (YL 9170 RI Detector), and column HPX-87C at 85 °C (BioRad Laboratories, Hercules, CA, USA), using water as the mobile phase with a flow rate of 0.6 mL/min. For the quantification of trisaccharides, raffinose was used as a standard. All standard substances were purchased from Merck KGaA, Darmstadt, Germany.

## 5. Conclusions

In this study, the β-galactosidase gene from the yogurt strain *L. bulgaricus* 43 was sequenced and found to encode a novel enzyme with 21 amino acid substitutions compared to all previously known β-galactosidases of this species. Through molecular modeling, it was shown that the structure of the enzyme suggests a tetrameric form, as well as a propensity for the formation of galactooligosaccharides with three monomers (DP3) and specific β-(1→4) and β-(1→3) linkages. The successful heterologous expression of the enzyme in *E. coli* strain BL21 (DE3) led to obtaining a recombinant enzyme with enormous activity (over 3000 U/mL, ~2010 U/mg) and the in vitro synthesis of 70.9 g/L of trisaccharides in the course of lactose conversion. Thus, the β-Gal of *L. bulgaricus* 43 is very promising for application to obtain GOS in industrial conditions.

## Figures and Tables

**Table 1:** Enzyme activity, GOS production, and influence of metal ions on specific enzyme activity (SEA) of selected microbial β-galactosidases.

Species, Strain	Type of Enzyme ^1^	Lactose (g/L)	GOS (g/L)	GOS (%) ^2^	SEA ^3^ (U/mg)	Metal Ions ^4^	Ref.
*L. bulgaricus* 43	Crude	200	70.91 (DP3)	34 (DP3)	2011 (O)	↑ Mn^2+^, Mg^2+^, Ca^2+^ ↓ Zn^2+^, Cu^2+^	This study
*L. bulgaricus* CRL450	Cell-free extract	300	n/a	41.3	2.06 (O)	n/a	[36]
*L. bulgaricus* DSM 20081	Purified, non-His-tag	205	102	50	317 (O) 123 (L)	↑ Na^+^, K^+^ ↓ Mg^2+^, Ca^2+^	[27]
*L. bulgaricus* wch9901	Crude	n/a	n/a	n/a	6.2 (O)	n/a	[35]
*L. acidophilus* R22	Purified natural	205	n/a	38.5	361 (O) 28.8 (L)	↑ Mg^2+^ ↓ Mn^2+^, Cu^2+^, Zn^2+^	[37]
*Lim. fermentum* K4	Purified	200-400	n/a	37	184 (O) 41 (L)	↑ Na^+^, K^+^, Mg^2+^	[38]
*L. helveticus* DSM 20075	Purified	205	n/a	n/a	476 (O) 11.1 (L)	↑ K^+^, Na^+^ ↓ Mn^2+^, Mg^2+^, Ca^2+^, Zn^2+^	[39]
*L. leichmannii* 313	Purified	n/a	n/a	n/a	31.28 (O)	↑ Na^+^ ↓ Ca^2+^, Mn^2+^	[35]
*Bifidobacterium breve* DSM 20213	Purified (2 enzymes)	200	n/a	33-44	489 (O) 59 (L)	n/a	[23]
*Bif. longum* *Bif. pseudocatenulatum*	Purified (2 enzymes)	n/a	n/a	n/a	2200 (O) 0.58 (O)	↑ Zn^2+^, Na^+^, Ca^2+^, Mn^2+^ ↓ Al^3+^	[40]
*B. circulans*	commercial	400	198	41	n/a	n/a	[34]
*Pyrococcus woesei*	Purified	n/a	n/a	n/a	5400 (O)	n/a	[41]
*Aspergillus oryzae*	commercial	400	107	26.8	n/a	n/a	[42]

^1^ All recombinant and His-tagged unless otherwise noted; ^2^ Percentage of total sugars; ^3^ SEA, specific enzyme activity (U/mg protein); substrates: O = ONPG; L = lactose; ^4^ Activators/inhibitors of SEA; ↑, activation; ↓, inhibition; 1U = μmol/min.

**Table 2:** Primers used in this study.

Primer	Sequence (5′-3′) ^1^	Positions in *β-gal* ^2^	Purpose
LacZ_F	ATGAGCAATAAGTTAGTAAAAGAAAAAAG	1–29	Sequencing
LacZ_R	TTATTTTAGTAAAAGGGGCTGAATCAC	3000–3027	Sequencing
LacZ_FF	GTGAAGGTGACTTGGTTGCTGAAAA	803–828	Sequencing
LacZ_RR	CCAGAAGGTAAATTCCGGCAGCCGCTTC	2285–2313	Sequencing
LacZ_*Nde*	CAGTCCATATGATGAGCAATAAGTTAGTAAAAGAAAAAAG	1–29	Cloning
LacZ_*Xho*	CTAGTCTCGAGTTTTAGTAAAAGGGGCTGAATCAC	3000–3024	Cloning

^1^ The underlined sequences are sites recognized by endonucleases. ^2^ NCBI GenBank acc. no OP.

## Data Availability

Not applicable.
